# The impact of older person’s frailty on the care-related quality of life of their informal caregiver over time: results from the TOPICS-MDS project

**DOI:** 10.1007/s11136-017-1606-5

**Published:** 2017-05-31

**Authors:** Marloes Oldenkamp, Mariët Hagedoorn, Rafael Wittek, Ronald Stolk, Nynke Smidt

**Affiliations:** 1 Department of Epidemiology, University Medical Center Groningen, University of Groningen, PO Box 30.001, 9700 RB Groningen, The Netherlands; 2 Department of Health Sciences, Health Psychology, University Medical Center Groningen, University of Groningen, PO Box 30.001, 9700 RB Groningen, The Netherlands; 3 Department of Sociology, University of Groningen, Grote Rozenstraat 31, 9712 TG Groningen, The Netherlands; 4 Department of Geriatrics, University Medical Center Groningen, University of Groningen, PO Box 30.001, 9700 RB Groningen, The Netherlands; 5 Present Address: The Netherlands Institute for Health Services Research (NIVEL), PO Box 1568, 3500 BN Utrecht, The Netherlands

**Keywords:** Informal care, Frailty, Caregiver burden, Quality of life, Longitudinal study

## Abstract

**Purpose:**

To examine the impact of changes in an older person’s frailty on the care-related quality of life of their informal caregiver.

**Methods:**

Five research projects in the TOPICS-MDS database with data of both older person and informal caregiver at baseline and after 12 months follow-up were selected. Frailty was measured in five health domains (functional limitations, psychological well-being, social functioning, health-related quality of life, self-rated health). Care-related quality of life was measured with the Care-Related Quality of Life instrument (CarerQoL-7D), containing two positive (fulfilment, perceived support) and five negative dimensions (relational problems, mental health problems, physical health problems, financial problems, problems combining informal care with daily activities).

**Results:**

660 older person/caregiver couples were included. Older persons were on average 79 (SD 6.9) years of age, and 61% was female. Caregivers were on average 65 (SD 12.6) years of age, and 68% was female. Results of the multivariable linear and logistic regression analyses showed that an increase in older person’s frailty over time was related to a lower total care-related quality of life of the caregiver, and to more mental and physical health problems, and problems with combining informal care with daily activities at follow-up. A change in the older person’s psychological well-being was most important for the caregiver’s care-related quality of life, compared to the other health domains.

**Conclusions:**

Health professionals observing decreasing psychological well-being of an older person and increasing hours of informal care provision should be aware of the considerable problems this may bring to their informal caregiver, and should tailor interventions to support informal caregivers according to their specific needs and problems.

**Electronic supplementary material:**

The online version of this article (doi:10.1007/s11136-017-1606-5) contains supplementary material, which is available to authorized users.

## Introduction

During the next two decades, the percentage of older persons with care needs will increase substantially. For the Netherlands, it is expected that the number of frail older persons aged 65 years and older will increase from 700.000 in 2010 to 1 million in 2030. Moreover, the share of people with multimorbidity (i.e. the presence of multiple chronic conditions) is expected to increase by more than 75% [1]; in turn, multimorbidity is associated with increases in care dependency [2]. Population aging poses an increasing challenge not only for policy makers and professionals in the health-care sector, but also for the growing group of informal caregivers. The provision of informal care poses a puzzle. On the one hand, informal care has clear advantages for society as a whole, as it is expected to reduce the use and costs of formal care. On the other hand, the provision of informal care has also consequences for burden, quality of life, and health of the informal caregiver.

Many informal caregivers experience their caregiving as enriching and satisfying, but they may also experience burden and stress at the same time [3]. The protection of caregiver quality of life and the prevention of caregiver burden is crucial, not only for caregivers themselves, but also to ensure a sufficient supply of informal care within the health-care system. High caregiver burden and low quality of life may lead to overburdened caregivers, health problems, and finally even to caregiver dropouts [4]. This has consequences for the informal caregiver him- or herself, such as increased health-care costs or negative impacts on labour-force participation [5, 6]. Moreover, health-care costs also increase because the care provided by informal caregivers needs to be taken over by others. The present study addresses the care-related quality of life experienced by informal caregivers [7, 8], and examines the impact of changes in the older person’s health problems on the care-related quality of life of informal caregivers.

The older person’s health problems have been associated with caregiver’s well-being and caregiver burden [9, 10]. However, evidence on how the care recipient’s health problems affect a caregiver’s well-being over time is inconsistent [11, 12]. On the one hand, an increase in the care recipient’s health problems has been related to an increase in caregiver burden and a decrease in caregiver quality of life over time. For example, an increase in the severity of functional limitations among dementia patients has been related to a decrease in caregiver’s psychological well-being [12], and an increase in the caregiver’s level of depressive symptoms [13]. These results are in line with the wear-and-tear hypothesis, which considers caregiving as a chronic stressor. It proposes that the accumulation of caregiving demands impairs the resources and well-being of the caregiver, leading to negative impacts of caregiving over time [14, 15]. On the other hand, other studies found no association between a care recipient’s deteriorating health and caregiver quality of life. This finding is more in line with the adaptation hypothesis, according to which caregivers learn to adapt to worsening health conditions of the care recipient. The negative impact of caregiving is the highest at the start of caregiving, but levels off or diminishes over time [15–17]. For example, a study among informal caregivers of older persons with health problems found that caregiver burden, measured with the Zarit Burden Interview, significantly decreased over time [11]. Interestingly, they found that the level of role strain decreased over time, while the level of personal strain remained constant. This suggests that while caregiving itself may remain stressful over time, caregivers may become more accustomed to their role as caregiver. Unfortunately, due to a small study population, the researchers were not able to examine which factors related to the changes in burden [11]. Other factors, such as changes in the hours of informal caregiving or available support need to be taken into account as well, as they may affect the associations between health problems of the care recipient and caregiver burden (see, for example [18]).

An explanation for the inconsistent results may be that studies not always specify different dimensions of caregiver burden, and focus on the negative outcomes of informal caregiving. Caregiver burden is a multidimensional concept and encompasses domain-specific (physical, psychological, financial, social) burdens [19–21]. However, informal caregivers may also experience beneficial aspects of caregiving, such as satisfaction or fulfilment [22]. In the current study, we focus on the care-related quality of life of informal caregivers, which includes both the negative and positive consequences of caregiving (i.e. care-related fulfilment, relational problems with the care recipient, mental health problems, physical health problems, problems completing daily activities, financial problems, and social support) [7, 8]. As such, it differs from caregiver burden, which is only negative in nature and concerns the evaluation of the care process itself. Both care-related quality of life and burden may affect a caregiver’s general well-being, but general well-being is also determined by other factors outside informal caregiving.

Taking into account the multiple dimensions of care-related quality of life may explain the inconsistent results concerning the relation between the older person’s health problems and caregiving outcomes over time. For instance, a deteriorating health may be related to more problems with completing daily activities because of increased informal care provision (wear-and-tear hypothesis). However, at the same time, being able to care for a loved one who deals with increasing health problems may bring more care-related fulfilment and feelings of enrichment (adaptation hypothesis).

To be able to include older persons with various diseases and illnesses, we use a measure of the care recipient’s health problems that can be applied to all persons, regardless of their diseases and illnesses: frailty. Frailty refers to a state of vulnerability to the experience of adverse health outcomes, and is based on the concept of deficit accumulation [23, 24]. These deficits in health can be based on a wide range of functional limitations, morbidities, or symptoms, all of which can be determined for each individual older person, regardless of their specific disease(s). By means of the older person’s level of frailty and changes in frailty over time, study results can be generalized to a large group of older persons and their informal caregivers.

This study examines the extent to which changes in an older person’s frailty over time (12 months period) influence the care-related quality of life experienced by their informal caregivers. In addition to frailty and care-related quality of life, multiple health domains of frailty (i.e. functional limitations, psychological well-being, social functioning, health-related quality of life, self-rated health) and multiple positive and negative dimensions of care-related quality of life are studied. This study thus improves our current knowledge on which changes in the specific health domains of frailty influence the different positive and negative dimensions of care-related quality of life of informal caregivers.

## Methods

### The Older Persons and Informal Caregivers Survey Minimum DataSet

Data from ‘The Older Persons and Informal Caregivers Survey Minimum DataSet’ (TOPICS-MDS) were used [25, 26]. TOPICS-MDS is a public data repository, developed to combine information from many research projects that were funded by the National Care for the Elderly Programme (Nationaal Programma Ouderenzorg), on behalf of the Organisation of Health Research and Development (ZonMw—The Netherlands). All research projects, although varying in study design, sampling frame, and inclusion criteria, used the same uniform and validated instruments measuring the physical, psychological, and social health and well-being of older persons and their informal caregivers [25, 26]. More detailed information about TOPICS-MDS and the individual research projects can be found elsewhere (www.topics-mds.eu) [25, 26].

### Selection of studies and respondents

The TOPICS-MDS database (TOPICS-MDS version 2, 2014) contains baseline data of 37.692 older persons and 3940 informal caregivers, originating from 42 research projects [25]. For our study, we selected research projects that included data of both older person and informal caregiver at baseline and after 12 months follow-up. A check was done on gender, age, and type of care relationship to ensure that the informal caregivers who participated at follow-up were the same as the informal caregivers who participated at baseline. Older persons who were living in a nursing home or home for the aged at baseline and/or follow-up were excluded from the dataset, because informal care provision for an older person living in a nursing home or home for the aged differs from informal care provision for a community-dwelling older person [27]. Finally, data from 5 research projects (3 prospective studies, 2 RCT’s) were used in the study, including 905 care recipient/caregiver couples. After exclusion of 198 (22%) caregivers with missing values on the outcome variables, and the exclusion of 47 (5%) caregivers with missing values on all variables related to the caregiver or caregiving situation, or all variables related to the health situation of the care recipient, the study population consisted of 660 care recipient/caregiver couples.

### Measurements

#### Care-related quality of life

Care-related quality of life was measured with the Care-Related Quality of Life instrument (CarerQoL) [8]. The CarerQoL instrument is a suitable measure of the individual experience of informal care provision, as it measures both the positive and negative impact of caregiving on the informal caregiver [7, 8]. It has been validated in different study designs, sampling frames and survey modes, using the TOPICS-MDS database [7]. Several heterogeneous caregiving samples showed its psychometric properties are good [8, 28]. The CarerQoL-7D describes the impact of caregiving on seven dimensions, including two positive dimensions (care-related fulfilment and perceived social support), and five negative dimensions (relational problems with the care recipient, mental health problems, problems combining daily activities, financial problems, and physical health problems). At both baseline and follow-up, caregivers described their personal care situation by indicating whether they had no, some, or a lot of problems for each dimension. Caregiver responses were dichotomized (combination of ‘no’ and ‘some’ for positive dimensions; combination of ‘some’ and ‘a lot’ for negative dimensions, due to low percentages). To calculate a single summary score based on the seven dimensions, a set of weights (a ‘tariff’) was applied to each level of each dimension [29]. The CarerQoL-7D summary score represents the overall care-related quality of life, in which both the negative and the positive impacts of caregiving are included, and ranges from 0 (worst care situation) to 100 (best care situation). In the statistical analyses the square root of the summary score at follow-up was used as the outcome variable, because of a moderately negative skewed distribution [30].

#### Frailty

Five health domains were used to construct a frailty index: (1) functional limitations [Katz Index of Independence Basic Activities of Daily Living (ADL), Instrumental Activities of Daily Living (IADL), and an additional indicator of mobility] (15 deficits) [31]; (2) psychological well-being (Rand-36 mental health subscale) (5 deficits) [32, 33]; (3) social functioning (single question derived from Rand-36) (1 deficit) [32, 33]; (4) health-related quality of life (EQ-5D+C) (6 deficits) [34]; (5) self-rated health (two questions from Rand-36) (2 deficits) [32, 33]. Based on the concept of deficits accumulation [24], the number of deficits on the included health domains was calculated (possible range 0–29), and divided by the total number of possible deficits (29 deficits). Deficits include a range of functional limitations, morbidities, or symptoms, such as needing help getting dressed (ADL) or having extreme pain or discomfort (EQ-5D+C). This resulted in a frailty index for each care recipient, ranging from 0 (no frailty) to 1 (extreme frailty). This frailty index was calculated for both baseline and follow-up, and a change score was calculated, taking into account floor- and ceiling effects (i.e. taking into account the highest and lowest possible scores) [35]. This change score ranged from −1 to +1, with a negative score indicating a decrease in frailty and a positive score indicating an increase in frailty. Change scores for the five health domains of frailty were calculated based on the scores at baseline and follow-up, taking into account floor- and ceiling effects. Negative scores indicated a decrease in the health domain (i.e. less functional limitations, lower psychological well-being), and positive scores indicated an increase in the health domain (i.e. more functional limitations, higher psychological well-being).

#### Covariates

The characteristics of the care recipient and caregiver consisted of age (in years) and gender (0 = male, 1 = female). Characteristics of the care situation were the type of care relationship (caring for a spouse, parent (in-law), someone else, or unknown/missing), whether or not care recipient and caregiver were living together and how this changed between baseline and follow-up, whether or not there was support available from another caregiver or volunteer and how this changed between baseline and follow-up, and changes in the total hours of informal care provision a week (continuous). The change score for total hours of care provision a week was calculated based on the scores at baseline and follow-up, taking into account floor- and ceiling effects, and ranged from −1 to +1. In addition, research project (dummies) and whether or not the care recipient and/or caregiver was allocated to an intervention (no/yes/unknown) were included.

### Statistical analyses

First, descriptive statistics were obtained for the study population characteristics (care recipient, caregiver, care situation) and the 5 research projects selected from the TOPICS-MDS database. Second, in order to examine the associations of changes in care recipient’s frailty and the health domains of frailty with the caregiver’s care-related quality of life at follow-up, uni- and multivariable linear regression analyses were conducted. The outcome variable was “total care-related quality of life of the caregiver at follow-up”. A statistical significance of .05 (*p* < .05) was used to test these associations. Third, in order to explore the associations of changes in frailty and the health domains of frailty with the positive and negative dimensions of care-related quality of life at follow-up, uni- and multivariable logistic regression analyses were conducted. The outcome variables were the different positive and negative dimensions of care-related quality of life at follow-up. A statistical significance of .01 (*p* < .01) was used, because of the many tests that were conducted with the positive and negative dimensions of care-related quality of life at follow-up as outcome. To take differences in the included research projects into account, all uni- and multivariable linear and logistic regression analyses were adjusted for research project (dummy for each research project) and whether or not the care recipient was allocated to an intervention (dummies yes/no/unknown). Furthermore, the uni- and multivariable linear and logistic regression analyses were adjusted for the baseline score of frailty, the baseline scores of the health domains of frailty, and the baseline score of total hours of informal care provision a week, if applicable (results of baseline scores not presented in the tables). Multicollinearity diagnostics were evaluated to check for multicollinearity in the multivariable models. If multicollinearity was evident (condition index >10.0 and variance proportions >.50), collinear variables were entered into separate regression models, and presented separately.

Due to a high percentage of missing values on some variables (see Online Resource Table 1), multiple imputation was applied for the missing values on continuous and dichotomous independent variables (fully conditional specification, predictive mean matching for imputation of continuous variables [36]). 39 datasets were created, with 100 iterations for each dataset, because 39% of all respondents had at least one missing value. The imputation model contained (a) all variables that were used in the analyses, including the outcome variables, as this gives more reliable results [37], (b) auxiliary variables that measured constructs comparable to variables in the analysis, and (c) informative variables related to research project (i.e. study design, sampling frame, intervention yes/no, research project). Missing values on the outcome variables were not imputed, because this may introduce noise to the estimates [38, 39]. As a result, analyses were conducted in the subset of respondents with complete data on care-related quality of life at follow-up (total score and dimensions). To facilitate convergence of the imputation model, missing values on categorical variables changes in living together of care recipient and caregiver and changes in available support from other caregiver/volunteer were not imputed, but instead an extra category ‘missing’ that contained the missing values was included. All statistical analyses were performed using IBM SPSS Statistics 23.

## Results

### Study population

Caregivers with missing values on the outcome variables and/or missing values on all variables related to the caregiver, caregiving situation, or health situation of the care recipient were excluded, reducing the study population from 905 to 660 care recipient/caregiver couples (see method section). Differences between included (*N* = 660) and excluded (*N* = 245) care recipient/caregiver couples (see Online Resource Table 2) show that included caregivers were younger, provided more hours of informal care a week, and had a lower care-related quality of life than excluded caregivers. In addition, the care recipients of included caregivers were older, more often their parent (in-law), and they had a higher frailty score, a lower psychological well-being and a lower self-rated health, compared to care recipients of excluded care recipients.

Characteristics of the study population and the care situation are presented in Table 1. The care recipient’s (changes in) frailty and health domains of frailty, and the caregiver’s total care-related quality of life and dimensions, are presented in Table 2. The average changes in frailty and in care-related quality of life between baseline and follow-up were small. Information about the research projects from which data was included, is presented in Online Resource Table 3.

**Table 1:** Characteristics of care recipient, caregiver, and care situation (*N* = 660)

	N (%)^a^
Care recipient characteristics	
Mean age (SD) (53–101)	79.1 (6.91)
Gender	
Male	256 (39%)
Female	404 (61%)
Caregiver characteristics	
Mean age (SD) (21–97)	64.6 (12.61)
Gender	
Male	208 (32%)
Female	452 (68%)
Care situation characteristics	
Type of care relationship (CG caring for)	
Spouse	331 (50%)
Parent (in-law)	265 (40%)
Other	64 (10%)
Older person and caregiver living together—baseline (T0)	
No	310 (47%)
Yes	346 (52%)
Missing	4 (1%)
Older person and caregiver living together—follow-up (T12)	
No	307 (47%)
Yes	350 (53%)
Missing	3 (0%)
Older person and caregiver living together—change between T0 and T12	
No–no	301 (46%)
Yes–yes	339 (51%)
No–yes	9 (1%)
Yes–no	4 (1%)
missing	7 (1%)
Support other caregiver/volunteer available—baseline (T0)	
No	461 (68%)
Yes	192 (31%)
Missing	11 (2%)
Support other caregiver/volunteer available—follow-up (T12)	
No	447 (68%)
Yes	202 (31%)
Missing	11 (2%)
Support other caregiver/volunteer available—change between T0 and T12	
No–no	368 (56%)
Yes–yes	114 (17%)
No–yes	86 (13%)
Yes–no	74 (11%)
Missing	18 (3%)
Total hours of informal care provision a week (0–168)—baseline (T0) (median, IQR)	8.0 (3.0–19.0)
Total hours of informal care provision a week (0–168)—follow-up (T12) (median, IQR)	9.0 (3.0–21.0)
Total hours of informal care provision a week—Change score T0–T12 (−1 to +1) (mean, SD)	−.20 (.35)

In this table non-imputed results are presented; the total N on continuous variables might differ because of missing values

*T0* baseline, *T12* follow-up, *SD* standard deviation, *IQR* interquartile range

^a^ *N*(%) are presented, unless indicated otherwise

**Table 2:** Health problems of care recipient and care-related quality of life of caregiver (*N* = 660)

Health problems of care recipient^a^	T0 (mean, SD)	T12 (mean, SD)	Change score (−1 to +1) (mean, SD)
Frailty (0–1)	.33 (.16)	.32 (.17)	−.09 (.26)
Functional limitations (0–15)	4.45 (3.25)	4.34 (3.32)	−.10 (.37)
Psychological well-being (0–100)	69.28 (17.98)	71.02 (17.45)	.13 (.33)
Social functioning (1–5)	3.71 (1.25)	3.76 (1.18)	.08 (.50)
Health-related quality of life (−.33–+1)	.61 (.28)	.66 (.27)	.16 (.39)
Self-rated health (1–5)	2.35 (.79)	2.31 (.70)	−.06 (.34)

Care-related quality of life caregiver	T0	T12
Total score (0–100) (median, IQR)	83.10 (73.9–89.6)	80.42 (74.0–90.0)
Dimensions (*N*, %)		
Fulfilment from caregiving (a lot)	388 (60%)	366 (55%)
Perceived support (a lot)	111 (17%)	69 (10%)
Relational problems (some/a lot)	230 (35%)	263 (40%)
Mental health problems (some/a lot)	294 (45%)	298 (45%)
Physical health problems (some/a lot)	363 (55%)	384 (58%)
Problems combining daily activities (some/a lot)	306 (47%)	283 (43%)
Financial problems (some/a lot)	57 (9%)	64 (10%)

In this table, the non-imputed results are presented, the total N on T0 variables might not be 660 due to missing values

*T0* baseline, *T12* follow-up, *SD* standard deviation

^a^Minimum and maximum scores are presented in parentheses

### Change in frailty and total care-related quality of life of the caregiver

Table 3 presents the results of the uni- and multivariable linear regression analyses examining the associations of the changes in care recipient’s frailty and health domains of frailty between baseline and follow-up with the caregiver’s total care-related quality of life at follow-up. An increase in frailty over time was related to a lower care-related quality of life of the caregiver in the univariable model. Inclusion of the caregiver’s baseline care-related quality of life and all other covariates to the model resulted in a smaller, and borderline statistically significant (*p* value .054) association between change in frailty and care-related quality of life at follow-up.

**Table 3:** Uni- and multivariable linear regression analyses with outcome of total care-related quality of life at T12 (*N* = 660)

	Univariable models			Multivariable model 1 (frailty)			Multivariable model 2 (frailty domains)		
	*b* (SE)	(95% CI)	*p*	*b* (SE)	(95% CI)	*p*	*b* (SE)	(95% CI)	*p*
Care recipient health changes T0–T12^a^									
Increase in frailty	−8.466 (3.088)	(−14.497; −2.435)	.006	−5.282 (2.742)^b^	(−10.657; .093)	.054	n.a.		
Increase in functional limitations	−3.505 (2.200)	(−7.809; .799)	.111	n.a.			−1.158 (2.069)	(−5.214; 2.898)	.576
Increase in psychological well-being	7.825 (2.329)	(3.260; 12.391)	.001	n.a.			4.259 (2.031)^b^	(.279; 8.240)	.036
Increase in social functioning	.502 (1.949)	(−3.338; 4.341)	.797	n.a.			−2.113 (1.647)	(−5.360; 1.133)	.201
Increase in health-related quality of life	6.626 (2.045)	(2.618; 10.634)	.001	n.a.			1.446 (2.007)	(−2.488; 5.380)	.471
Increase in self-rated health	7.510 (2.421)	(5.089; 9.931)	.002	n.a.			2.417 (2.157)	(−1.811; 6.645)	.263
Care recipient characteristics									
Age	.097 (.123)	(−.145; .338)	.433	.118 (.128)	(−.133; .369)	.358	.115 (.131)	(−.141; .371)	.379
Female	4.252 (1.601)	(1.115; 7.390)	.008	−.231 (1.705)	(−3.572; 3.110)	.892	−.083 (1.718)	(−3.451; 3.284)	.961
Caregiver characteristics									
Age	−.208 (.061)	(−.329; −.088)	.001	−.142 (.094)	(−.327; .043)	.131	−.143 (.094)	(−.328; .041)	.127
Female	−2.571 (1.656)	(−5.817; .674)	.120	−2.929 (1.805)^b^	(−6.468; .610)	.105	−2.904 (1.802)^b^	(−6.436; .628)	.107
Total care-related quality of life T0	.887 (.047)	(.794; .980)	.000	.859 (.049)	(.762; .956)	.000	.850 (.050)	(.752; .947)	.000
Care situation characteristics									
Type of care relationship (caring for)									
Spouse	Ref.			Ref.			Ref.		
Parent (in-law)	5.996 (1.701)	(2.661; 9.330)	.000	−3.619 (4.144)	(−11.742; 4.503)	.383	−4.041 (4.148)	(−12.172; 4.090)	.330
Other	6.670 (2.745)	(1.290; 12.050)	.015	−4.335 (4.071)	(−12.314; 3.644)	.287	−4.567 (4.077)	(−12.558; 3.424)	.263
Living together									
T0: no, T12: no	Ref.			Ref.			Ref.		
T0: yes, T12: yes	−6.855 (1.653)	(−10.094; −3.616)	.000	−3.282 (3.499)	(−10.140; 3.576)	.348	−3.946 (3.505)^b^	(−10.816; 2.924)	.260
T0: no, T12: yes	6.045 (6.670)	(−7.028; 19.119)	.365	6.781 (5.466)	(−3.931; 7.494)	.215	5.404 (5.515)	(−5.405; 16.213)	.327
T0: yes, T12: no	6.054 (9.801)	(−13.156; 25.263)	.537	1.306 (8.289)	(−14.940; 17.552)	.875	1.941 (8.320)	(−14.366; 18.248)	.816
unknown/missing	3.988 (7.476)	(−10.665; 18.641)	.594	12.068 (6.561)	(−.793; 24.928)	.066	11.248 (6.597)	(−1.682; 24.178)	.088
Support other caregiver/volunteer available									
T0: no, T12: no	Ref.			Ref.			Ref.		
T0: yes, T12: yes	1.599 (2.127)	(−2.571; 5.768)	.452	.673 (1.804)	(−2.863; 4.209)	.709	.162 (1.852)	(−3.468; 3.793)	.930
T0: no, T12: yes	−.322 (2.389)	(−5.004; 4.360)	.893	1.152 (1.988)	(−2.744; 5.047)	.562	1.279 (1.987)	(−2.617; 5.174)	.520
T0: yes, T12: no	−1.146 (2.515)	(−6.074; 3.783)	.649	−2.391 (2.080)	(−6.467; 1.685)	.250	−2.287 (2.096)	(−6.396; 1.821)	.275
unknown/missing	−.585 (4.835)	(.110; 19.061)	.047	9.429 (3.929)^b^	(1.728; 17.130)	.016	9.326 (3.964)	(1.556; 17.096)	.019
Change total hours informal care provision T0–T12^a^	−5.369 (2.417)	(−10.110; −.629)	.026	−4.487 (2.112)	(−8.631; −.343)	.034	−4.653 (2.130)	(−8.833; -.473)	.029

All uni- and multivariable models are adjusted for research project and intervention (yes/no/unknown)

The quadratic term of total care-related quality of life at T12 is used, because of skewed distribution

*T0* baseline, *T12* follow-up, *n.a.* not applicable, *SE* standard error, *95% CI* 95% confidence interval; a significance level of *p* < .05 is used

^a^The uni- and multivariable models are adjusted for the baseline score of the health of the care recipient (i.e. frailty, functional limitations, etc.), and are adjusted for the baseline score of total hours of informal care provision

^b^Statistically significant (*p* < .05) in model without adjustment for total care-related quality of life at T0

### Changes in health domains of frailty and total care-related quality of life of the caregiver

A closer look at changes in the health domains of frailty over time (Table 3, multivariable model 2) showed that an increase in the psychological well-being of the care recipient was related to a higher care-related quality of life of the caregiver. No statistically significant associations were found between changes in the other health domains of frailty (i.e. functional limitations, social functioning, health-related quality of life, self-rated health) and the total care-related quality of life of the caregiver at follow-up. The association between a change in the hours of informal care provision and care-related quality of life was statistically significant, suggesting that informal caregivers whose hours of informal care provision increased between baseline and follow-up experience a lower care-related quality of life at follow-up. No statistically significant associations were found for the other covariates, including type of care relationship, changes in whether care recipient and caregiver were living together, and changes in the available support. Except for the category ‘unknown/missing’ of the variable changes in available support, suggesting that caregivers with unknown changes in available support experienced a higher total care-related quality of life at follow-up, compared to caregivers who had no support available at baseline and follow-up.

### Change in frailty and the dimensions of care-related quality of life of the caregiver

Online Resource Tables 4–10 present the results of the uni- and multivariable logistic regression analyses examining the associations between changes in care recipient’s frailty and health domains of frailty between baseline and follow up with the seven dimensions of the caregiver’s care-related quality of life at follow-up. An increase in frailty over time was related to more mental health problems (Online Resource Table 7), more physical health problems (Online Resource Table 8), and more problems with combining informal care with other daily activities (Online Resource Table 9) for the caregiver at follow-up. A change in frailty over time was not related to fulfilment from caregiving (Online Resource Table 4), perceived support (Online Resource Table 5), relational problems (Online Resource Table 6), or financial problems (Online Resource Table 10) of the caregiver at follow-up.

### Changes in health domains of frailty and the dimensions of care-related quality of life of the caregiver

With regard to changes in the health domains of frailty over time, we found that an increase in the psychological well-being of the care recipient over time was related to fewer mental health problems, but with a *p*-value of .033 (Online Resource Table 7) for the caregiver at follow-up. In addition, an increase in care recipient’s self-rated health over time was related to fewer problems with combining daily activities (Online Resource Table 9). None of the health domains of frailty were related to fulfilment from caregiving, perceived support, relational problems, physical health problems, or financial problems at follow-up. Furthermore, no statistically significant associations were found between changes in care recipient’s functional limitations, social functioning, or health-related quality of life and the positive and negative dimensions of the caregiver’s care-related quality of life at follow-up. Caregivers with support available at follow-up but not at baseline experienced more relational problems at follow-up (vs. no support available at baseline and follow-up) (Online Resource Table 6). When the total hours of informal care provision increased between baseline and follow-up, informal caregivers experienced more relational problems at follow up (Online Resource Table 6). Older caregivers experienced more physical health problems at follow-up (Online Resource Table 9).

## Discussion

The ageing population poses an increasing challenge for policy makers, health-care professionals, and informal caregivers. The care recipient’s health situation has an impact on the well-being of informal caregivers [9, 10], but evidence on how changes in this health situation over time affect the informal caregiver is inconsistent [11, 12]. This study suggested that an increase in frailty of the care recipient over a 12-month period relates to a lower care-related quality of life of the informal caregiver at follow-up, which is particularly reflected in more mental health problems, more physical health problems, and more problems with the combination of their informal care tasks with other daily activities. The finding that increased frailty did not affect fulfilment from caregiving and perceived support from others, e.g. positive caregiving experiences, corresponds with previous research demonstrating that positive and negative caregiving experiences are two different concepts, with different predictors [40–44].

More interestingly, changes in the care recipient’s psychological well-being, one of the included health domains of frailty, turned out to be important. Caregivers whose care recipient’s psychological well-being increased over time, experienced a higher overall care-related quality of life at follow-up, and also seemed to experience fewer mental health problems due to caregiving. An explanation for the importance of changes in psychological well-being could be that a decline in psychological well-being may also introduce communication problems like difficulties with understanding each other, or behaviour problems such as demanding or difficult behaviours or emotional lability. As such, these changes may make caregivers feel helpless in their situation as informal caregiver and make the psychological adjustment more difficult [10]. This may particularly affect the experience of mental health problems, as suggested in this study. Moreover, previous studies suggest that care recipient’s behavioural problems have stronger associations with caregiver outcomes such as burden and depression than other stressors like physical impairments [10]. More research is needed to increase our understanding of the importance of psychological well-being and communication problems for the informal caregiver’s care-related quality of life.

The results suggest that the care-related quality of life of the caregiver fluctuates along with changes in the health of the care recipient, particularly with changes in psychological well-being negatively affecting mental health problems of the informal caregiver and changes in self-rated health affecting the experience of problems with combining daily activities. This seems to be in line with the wear-and-tear hypothesis, in which a deterioration of the care recipient’s health is expected to negatively impact a caregiver’s well-being [14, 15]. Moreover, the finding that an increase in hours of informal caregiving between baseline and follow-up negatively affected the informal caregiver’s care-related quality at follow-up also supports the wear-and-tear hypothesis. However, these conclusions need to be seen in a nuanced light, because the wear-and-tear hypothesis was not applicable to all health domains of frailty and all dimensions of care-related quality of life. For instance, increasing functional limitations did not relate to any of the dimensions of care-related quality of life, in contrast to psychological well-being. We did not find any associations between changes in psychological well-being, or changes in one of the other health domains of frailty, and the experience of fulfilment from caregiving. The existence of these associations would be in line with the adaptation hypothesis, which proposes that caregivers adapt to the situation despite a deterioration of the care recipient’s health [15–17]. However, we did not found such associations. Our findings also suggest a third conclusion: caregiver care-related quality of life seems to be subject to situational fluctuations, in particular related to (changes in) the mental health of the care recipient and changes in the hours of informal care provision. More research is necessary on wear-and-tear, adaptation, and changes in the care recipient’s health and caregiver’s care-related quality of life over time. This study showed that for better and more precise insights in the relation between changes in a care recipient’s health and caregiver’s care-related quality of life, it is necessary to divide care-related quality of life into different dimensions and to distinguish different health domains of a care recipient’s health situation.

This study has some limitations that need to be mentioned. First, in several of the research projects that provided data for this study, a fairly large group of informal caregivers and/or care recipients dropped out after the baseline measurement. Unfortunately, there is no information available about the reasons for drop-out. Selection bias might have been introduced into our study, because reasons for drop-out may have been related to health problems of the care recipient and/or care-related quality of life of the informal caregiver. Second, we excluded 245 informal caregivers/care recipients with missing values on all variables related to the caregiver/caregiving situation, all variables related to the health situation of the care recipient, or all variables related to the care-related quality of life of the caregiver. The results of this study need to be interpreted with some caution, because differences between the in- and excluded caregiver/care recipient couples exist on multiple characteristic. Included caregivers cared for care recipients with higher frailty scores, had a lower level of care-related quality of life and provided more hours of informal care a week. Particularly indicators of the level of caregiving involvement, such as hours of caregiving, may be stronger related to negative caregiving outcomes like caregiver burden among response caregivers, compared to nonresponding caregivers, as suggested by other caregiving research [45]. Third, only two measurement points with 12 months in between were included. Future research studying the impact of changes in a care recipient’s health on caregiver’s experiences and well-being could include more measurement points and a longer period of follow-up. In this way, nonlinear trajectories can be studied and better insights in the effect of fluctuations in the care recipient’s health over time can be created. Fourth, data of multiple different research projects was combined in this study. Although these research projects all used standardized and validated measures, they varied in study design and sampling frames. We adjusted for these differences by including dummy variables for research project and allocation to intervention in the statistical analyses, instead of multilevel modelling, because we were interested in individual caregiving experiences, and not so much in differences between research projects. The TOPICS-MDS database is an important and highly relevant initiative for the further development of caregiving research [25, 26]. Particularly the availability of information about the care recipients in combination with information about their informal caregivers provides interesting research possibilities.

To conclude, this study showed that a deteriorating health situation of older persons (i.e. an increase in frailty) has a negative impact on the care-related quality of life of their informal caregivers. Due to population ageing, people are expected to live longer at home, leading to an increasing demand for help from informal caregivers. Therefore, informal care may be provided more often in situations in which deteriorating health problems of care recipients also lead to declines in the well-being of informal caregivers. Health professionals observing increasing frailty and decreasing psychological well-being of an older person should be aware of the considerable problems this may bring to the older person’s informal caregiver. Caregiver interventions to support the informal caregiver should be discussed, for example respite care, which can offer a substitute for the informal care and provides a temporary relief to the caregiver, or support groups, providing opportunities to share personal feelings and concerns [46]. Because most interventions have domain-specific outcomes, health professionals have to tailor interventions according to the specific needs, stressors, resources, and problems of the informal caregiver [47].

## Electronic supplementary material

Below is the link to the electronic supplementary material.

Supplementary material 1 (DOCX 94 kb)
