# Supplementary material for: The impact of older person’s frailty on the care-related quality of life of their informal caregiver over time: results from the TOPICS-MDS project
Source: Qual Life Res. 2017 May 31;26(10):2705–16. doi: 10.1007/s11136-017-1606-5 (PMC5597689; doi:10.1007/s11136-017-1606-5)
Supplement: Supplementary file 1 — Supplementary material 1 (DOCX 94 kb) [file 11136_2017_1606_MOESM1_ESM.docx]

**Supplementary Material, Table 1 – 10**

Article title: The impact of older person’s frailty on the care-related quality of life of their informal caregiver over time: results from the TOPICS-MDS project

Journal: Quality of Life Research

Authors: Marloes Oldenkamp, Mariët Hagedoorn, Rafael Wittek, Ronald Stolk, Nynke Smidt

Corresponding author: Marloes Oldenkamp ([m.oldenkamp@nivel.nl](mailto:m.oldenkamp@nivel.nl))

| **Table 1: Overview of missing values per variable (N=660)** | |
| --- | --- |
|  | **Missing values**  **N (%)** |
| **Care recipient characteristics** |  |
| Mean age | 0 (0%) ^a^ |
| Gender | 0 (0%) ^a^ |
|  |  |
| **Caregiver characteristics** |  |
| Mean age | 0 (0%) ^a^ |
| Gender | 0 (0%) ^a^ |
|  |  |
| **Care situation characteristics** |  |
| Type of care relationship (CG caring for) | 0 (0%) ^a^ |
| Older person and caregiver living together – change between T0 – T12 | 7 (1.1%) ^b^ |
| Support other caregiver/volunteer available – change between T0 – T12 | 18 (2.7%) ^b^ |
| Total hours of informal care provision a week – change between T0 – T12 | 108 (16.4%) |
|  |  |
| **Health problems care recipient, change in…** |  |
| Frailty | 1 (0.2%) |
| Functional limitations | 1 (0.2%) |
| Psychological well-being | 16 (2.4%) |
| Social functioning | 184 (27.9%) |
| Health-related quality of life | 9 (1.4%) |
| Self-rated health | 2 (0.3%) |
|  |  |
| **Care-related quality of life caregiver T0** |  |
| Total score | 46 (7.0%) |
| Fulfilment from caregiving | 11 (1.7%) |
| Perceived support | 5 (0.8%) |
| Relational problems | 8 (1.2%) |
| Mental health problems | 8 (1.2%) |
| Physical health problems | 4 (0.6%) |
| Problems combining daily activities | 5 (0.8%) |
| Financial problems | 4 (0.6%) |
| T0 = baseline; T12 = follow-up  ^a^ not imputed, because no missing values  ^b^ missing values on these variables are not imputed, but included in the analyses as a separate category. | |

| **Table 2: Comparison of included (N=660) and excluded respondents (N=245) ^a^** | | | |
| --- | --- | --- | --- |
|  | **Included respondents (N=660) ^b,c^** | **Excluded respondents (N=245) ^c^** | **p-value ^d^** |
| **Care recipient characteristics** |  |  |  |
| Mean age (sd) (53-101) | 79.1 (6.91) | 76.7 (6.70) | .000 |
| Gender = female | 61% | 56% | .183 |
|  |  |  |  |
| **Caregiver characteristics** |  |  |  |
| Mean age (sd) (21-97) | 64.6 (12.61) | 66.9 (12.52) | .014 |
| Gender = female | 68% | 62% | .087 |
|  |  |  |  |
| **Care situation characteristics** |  |  |  |
| Type of care relationship (CG caring for)  - Spouse  - Parent (in-law)  - Other | 50%  40%  10% | 62%  29%  9% | .005 |
| Older person and caregiver living together – baseline (T0) = yes | 53% | 62% | .011 |
| Support other caregiver/volunteer available – baseline (T0) = yes | 29% | 29% | .989 |
| Total hours of informal care provision a week (0-168) – baseline (T0) (median, IQR) | 8.0 (3.0-20.0) | 2.0 (0.0-14.0) | .000 |
|  |  |  |  |
| **Health problems care recipient at baseline (T0)** |  |  |  |
| Frailty (0-1) | .33 (.16) | .30 (.14) | .008 |
| Functional limitations (0-15) | 4.45 (3.25) | 4.19 (3.02) | .284 |
| Psychological well-being (0-100) | 69.34 (17.94) | 76.26 (17.45) | .000 |
| Social functioning (1-5) | 3.63 (1.31) | 3.71 (1.37) | .455 |
| Health-related quality of life (-.33-1) | .61 (.29) | .62 (.27) | .638 |
| Self-rated health (1-5) | 2.35 (.79) | 2.73 (.88) | .000 |
|  |  |  |  |
| **Care-related quality of life caregiver at baseline (T0)** |  |  |  |
| Total score (0-100) (median, IQR) | 83.10 (73.9-89.6) | 87.10 (77.5-93.6) | .004 |
| Fulfilment from caregiving = a lot | 60% | 64% | .438 |
| Perceived support = a lot | 17% | 23% | .154 |
| Relational problems = some/a lot | 35% | 15% | .000 |
| Mental health problems = some/a lot | 45% | 28% | .001 |
| Physical health problems = some/a lot | 55% | 45% | .054 |
| Problems combining daily activities = some/a lot | 47% | 34% | .019 |
| Financial problems = some/a lot | 9% | 8% | .736 |
| T12 = follow-up, sd = standard deviation, IQR = interquartile range  ^a^ total N might differ because of missing values.  ^b^ Percentages might differ from Table 1 because missing categories are not included and presented in the current table.  ^c^ N(%) are presented, unless indicated otherwise  ^d^ Chi-square test for normally distributed categorical variables, Independent Samples T-test for normally distributed continuous variables, Mann-Whitney Test for not normally distributed variables. | | | |

| **Table 3: characteristics of research projects** | | | | |
| --- | --- | --- | --- | --- |
|  | **N (%)** | **Study design** | **Sampling frame** | **N in intervention** |
| Study 1 | 146 (22%) | Prospective | General population and nursing home | n.a. |
| Study 4 | 170 (26%) | Prospective | Hospital | n.a. |
| Study 10 | 143 (22%) | RCT | Primary care | 68 (40%) |
| Study 13 | 160 (24%) | RCT | Primary care | 116 (73%) |
| Study 21 | 41 (6%) | Prospective | Hospital | n.a. |
| n.a. = not applicable | | | | |

| **Table 4: Uni- and multivariable logistic regression analyses with outcome fulfilment from caregiving at T12 (N=660) ^a^** | | | | | | | | | |
| --- | --- | --- | --- | --- | --- | --- | --- | --- | --- |
| **Fulfilment from caregiving** | **Univariable models** | | | **Multivariable model 1 (frailty)** | | | **Multivariable model 2 (frailty domains)** | | |
| **0=no/some (45%), 1=a lot (55%)** | **OR** | **(95% CI)** | **p** | **OR** | **(95% CI)** | **p** | **OR** | **(95% CI)** | **p** |
| **Care recipient health changes T0-T12 ^b^** |  |  |  |  |  |  |  |  |  |
| Increase in frailty | 1.209 | (.646-2.264) | .552 | 1.681 | (.758-3.727) | .201 | n.a. |  |  |
| Increase in functional limitations | 1.439 | (.922-2.245) | .109 | n.a. |  |  | 1.764 | (.962-3.237) | .067 |
| Increase in psychological well-being | 1.248 | (.776-2.009) | .361 | n.a. |  |  | .919 | (.506-1.669) | .782 |
| Increase in social functioning | .971 | (.686-1.374) | .867 | n.a. |  |  | .812 | (.514-1.283) | .372 |
| Increase in health-related quality of life | 1.198 | (.788-1.821) | .398 | n.a. |  |  | 1.483 | (.820-2.682) | .192 |
| Increase in self-rated health | 1.505 | (.926-2.446) | .102 | n.a. |  |  | 1.282 | (.683-2.405) | .440 |
| **Care recipient characteristics** |  |  |  |  |  |  |  |  |  |
| Age | 1.004 | (.979-1.029) | .764 | 1.009 | (.972-1.047) | .651 | 1.007 | (.969-1.047) | .724 |
| Female | .963 | (.697-1.331) | .821 | 1.028 | (.631-1.676) | .910 | 1.028 | (.624-1.695) | .913 |
| **Caregiver characteristics** |  |  |  |  |  |  |  |  |  |
| Age | 1.002 | (.990-1.015) | .696 | .995 | (.968-1.024) | .750 | .996 | (.968-1.025) | .774 |
| Female | .989 | (.708-1.379) | .946 | .803 | (.479-1.346) | .404 | .792 | (.467-1.344) | .387 |
| Fulfilment T0 (ref. no/some) | 8.022 | (5.589-11.515) | .000 | 8.714 | (5.956-12.748) | .000 | 9.122 | (6.165-13.496) | .000 |
| **Care situation characteristics** |  |  |  |  |  |  |  |  |  |
| Type of care relationship (caring for)  - Spouse  - Parent (in-law)  - Other | ref.  .879  .946 | (.623-1.241)  (.543-1.650) | .464 .846 | ref.  .527  .537 | (.150-1.858)  (.157-1.831) | .319  .320 | ref.  .510  .511 | (.139-1.867)  (.144-1.813) | .309  .299 |
| Living together  - T0: no, T12: no  - T0: yes, T12: yes  - T0: no, T12: yes  - T0: yes, T12: no  - unknown/missing | ref.  1.069  6.897  2.763  2.498 | (.764-1.497)  (.841-56.598)  (.282-27.081)  (.473-13.177) | .695  .072  .383  .281 | ref.  .632  6.143  2.079  3.645 | (.219-1.824)  (.646-58.378)  (.167-25.900)  (.467-28.436) | .396  .114  .570  .217 | ref.  .605  5.222  2.036  3.062 | (.201-1.823)  (.549-49.656)  (.133-31.069)  (.365-25.727) | .372  .150  .609  .303 |
| Support other caregiver/volunteer available  - T0: no, T12: no  - T0: yes, T12: yes  - T0: no, T12: yes  - T0: yes, T12: no  - unknown/missing | ref.  .864  .635  .651  .996 | (.563-1.327)  (.393-1.025)  (.393-1.08)  (.378-2.628) | .505  .063  .095  .994 | ref.  1.036  .652  .769  1.724 | (.613-1.753)  (.367-1.157)  (.420-1.410)  (.560-5.301) | .894  .144  .396  .342 | ref.  .982  .643  .747  1.386 | (.570-1.690)  (.360-1.150)  (.404-1.383)  (.445-4.317) | .946  .136  .353  .573 |
| Change total hours informal care provision T0-T12 ^b^ | .718 | (.428-1.204) | .209 | .669 | (.353-1.266) | .216 | .648 | (.336-1.249) | .195 |
| T0 = baseline, T12 = follow-up, n.a. = not applicable, OR = Odds Ratio, 95% CI = 95% Confidence Interval; a significance level of p < .01 is used.  ^a^ all uni- and multivariable models are adjusted for research project and intervention (yes/no/unknown).  ^b^ all uni- and multivariable models are adjusted for baseline frailty and baseline frailty domains of the care recipient, and for the baseline total hours of informal care provision a week.  ^c^ statistically significant (p <.01) in model without adjustment for fulfilment at T0. | | | | | | | | | |

| **Table 5: Uni- and multivariable logistic regression analyses with outcome perceived support at T12 (N=660) ^a^** | | | | | | | | | |
| --- | --- | --- | --- | --- | --- | --- | --- | --- | --- |
| **Perceived support** | **Univariable models** | | | **Multivariable model 1 (frailty)** | | | **Multivariable model 2 (frailty domains)** | | |
| **0=no/some (90%), 1=a lot (10%)** | **OR** | **(95% CI)** | **p** | **OR** | **(95% CI)** | **p** | **OR** | **(95% CI)** | **p** |
| **Care recipient health changes T0-T12** |  |  |  |  |  |  |  |  |  |
| Increase in frailty | 2.083 | (.745-5.824) | .162 | 1.123 | (.329-3.834) | .854 | n.a. |  |  |
| Increase in functional limitations | 1.323 | (.644-2.720) | .446 | n.a. |  |  | .547 | (.209-1.429) | .218 |
| Increase in psychological well-being | .632 | (.286-1.400) | .258 | n.a. |  |  | .595 | (.238-1.488) | .267 |
| Increase in social functioning | 1.064 | (.558-2.031) | .849 | n.a. |  |  | 1.257 | (.595-2.657) | .547 |
| Increase in health-related quality of life | .573 | (.287-1.145) | .115 | n.a. |  |  | .731 | (.292-1.829) | .503 |
| Increase in self-rated health | .946 | (.635-1.409) | .889 | n.a. |  |  | 1.366 | (.527-3.538) | .521 |
|  |  |  |  |  |  |  |  |  |  |
| **Care recipient characteristics** |  |  |  |  |  |  |  |  |  |
| Age | 1.043 | (1.003-1.085) | .034 | 1.025 | (.969-1.084) | .389 | 1.035 | (.976-1.098) | .253 |
| Female | 1.686 | (.962-2.952) | .068 | 1.457 | (.662-3.209) | .350 | 1.417 | (.630-3.188) | .400 |
|  |  |  |  |  |  |  |  |  |  |
| **Caregiver characteristics** |  |  |  |  |  |  |  |  |  |
| Age | .984 | (.964-1.004) | .113 | .994 | (.954-1.035) | .350 | .992 | (.951-1.035) | .707 |
| Female | 1.184 | (.681-2.057) | .549 | 1.121 | (.507-2.481) | .777 | 1.090 | (.489-2.431) | .832 |
| Perceived support T0 (ref. no/some) | 7.601 | (4.426-13.054) | .000 | 5.465 | (3.025-9.873) | .000 | 5.978 | (3.221-11.091) | .000 |
|  |  |  |  |  |  |  |  |  |  |
| **Care situation characteristics** |  |  |  |  |  |  |  |  |  |
| Type of care relationship (caring for)  - Spouse  - Parent (in-law)  - Other | ref.  1.816  1.499 | (1.030-3.204)  (.605-3.715) | .039  .382 | ref.  .125  .161 | (.008-1.871)  (.010-2.532) | .132  .194 | ref.  .134  .162 | (.009-1.954)  (.010-2.525) | .142  .194 |
| Living together  - T0: no, T12: no  - T0: yes, T12: yes  - T0: no, T12: yes  - T0: yes, T12: no  - unknown/missing | ref.  .464  ^d^  ^d^  ^d^ | (.264-.814) | .007 | ref.  .120  ^d^  ^d^  ^d^ | (.008-1.696) | .117 | ref.  .137  ^d^  ^d^  ^d^ | (.010-1.848) | .134 |
| Support other caregiver/volunteer available  - T0: no, T12: no  - T0: yes, T12: yes  - T0: no, T12: yes  - T0: yes, T12: no  - unknown/missing | ref.  4.422  2.767  1.646  .856 | (2.369-8.254)  (1.322-5.791)  (.675-4.016)  (.107-6.865) | .000  .007  .273  .883 | ref.  2.406^c^  2.047  1.325  .902 | (1.166-4.965)  (.883-4.746)  (.490-3.582)  (.099-8.238) | .017  .095  .579  .927 | ref.  2.670^c^  2.282  1.236  .950 | (1.248-5.713)  (.972-5.355)  (.442-3.462)  (.100-9.000) | .011  .058  .686  .965 |
| Change total hours informal care provision T0-T12 ^b^ | .584 | (.275-1.242) | .162 | .668 | (.272-1.636) | .377 | .702 | (.282-1.745) | .446 |
| T0 = baseline, T12 = follow-up, n.a. = not applicable, OR = Odds Ratio, 95% CI = 95% Confidence Interval; a significance level of p < .01 is used.  ^a^ all uni- and multivariable models are adjusted for research project and intervention (yes/no/unknown).  ^b^ all uni- and multivariable models are adjusted for baseline frailty and baseline frailty domains of the care recipient, and for the baseline total hours of informal care provision a week.  ^c^ statistically significant (p <.01) in model without adjustment for perceived support at T0.  ^d^ not able to calculate due to small numbers of respondents. | | | | | | | | | |

| **Table 6: Uni- and multivariable logistic regression analyses with outcome relational problems at T12 (N=660) ^a^** | | | | | | | | | |
| --- | --- | --- | --- | --- | --- | --- | --- | --- | --- |
| **Relational problems** | **Univariable models** | | | **Multivariable model 1 (frailty)** | | | **Multivariable model 2 (frailty domains)** | | |
| **0=no (60%), 1=some/a lot (40%)** | **OR** | **(95% CI)** | **p** | **OR** | **(95% CI)** | **p** | **OR** | **(95% CI)** | **p** |
| **Care recipient health changes T0-T12** |  |  |  |  |  |  |  |  |  |
| Increase in frailty | 3.067 | (1.524-6.170) | .002 | 1.725 | (.740-4.022) | .207 | n.a. |  |  |
| Increase in functional limitations | 1.684 | (1.038-2.732) | .035 | n.a. |  |  | 1.152 | (.608-2.184) | .665 |
| Increase in psychological well-being | .427 | (.253-.722) | .001 | n.a. |  |  | .600 | (.318-1.131) | .114 |
| Increase in social functioning | .702 | (.482-1.023) | .065 | n.a. |  |  | .900 | (.570-1.420) | .650 |
| Increase in health-related quality of life | .538 | (.345-.839) | .006 | n.a. |  |  | 1.277 | (.694-2.350) | .432 |
| Increase in self-rated health | .481 | (.330-.701) | .005 | n.a. |  |  | .784 | (.404-1.522) | .472 |
|  |  |  |  |  |  |  |  |  |  |
| **Care recipient characteristics** |  |  |  |  |  |  |  |  |  |
| Age | 1.005 | (.980-1.031) | .677 | .982 | (.945-1.021) | .368 | .982 | (.944-1.022) | .367 |
| Female | .917 | (.658-1.276) | .606 | .981 | (.586-1.643) | .942 | 1.002 | (.592-1.696) | .993 |
|  |  |  |  |  |  |  |  |  |  |
| **Caregiver characteristics** |  |  |  |  |  |  |  |  |  |
| Age | 1.003 | (.990-1.015) | .698 | 1.000 | (.971-1029) | .981 | .999 | (.970-1.028) | .599 |
| Female | 1.095 | (.778-1.543) | .602 | 1.170 | (.683-2.004) | .567 | 1.157 | (.672-1.992) | .599 |
| Relational problems T0 (ref. no) | 8.408 | (5.801-12.186) | .000 | 8.483 | (5.632-12.777) | .000 | 8.781 | (5.762-13.380) | .000 |
|  |  |  |  |  |  |  |  |  |  |
| **Care situation characteristics** |  |  |  |  |  |  |  |  |  |
| Type of care relationship (caring for)  - Spouse  - Parent (in-law)  - Other | ref.  .965  .763 | (.679-1.372)  (.429-1.357) | .844  .357 | ref.  2.690  2.553 | (.714-10.139)  (.703-9.271) | .144  .154 | ref.  2.687  2.620 | (.697-10.364)  (.704-9.750) | .151  .151 |
| Living together  - T0: no, T12: no  - T0: yes, T12: yes  - T0: no, T12: yes  - T0: yes, T12: no  - unknown/missing | ref.  1.263  ^d^  ^d^  ^d^ | (.894-1.784) | .185 | ref.  2.346  ^d^  ^d^  ^d^ | (.754-7.304) | .141 | ref.  2.350  ^d^  ^d^  ^d^ | (.736-7.509) | .149 |
| Support other caregiver/volunteer available  - T0: no, T12: no  - T0: yes, T12: yes  - T0: no, T12: yes  - T0: yes, T12: no  - unknown/missing | ref.  1.292  2.378  1.544  .988 | (.834-2.000)  (1.641-3.873)  (.924-2.578)  (.365-2.670) | .251  .000  .097  .981 | ref.  1.047  2.317^c^  1.526  .973 | (.614-1.787)  (1.274-4.214)  (.824-2.829)  (.296-3.206) | .865  .006  .179  .965 | ref.  1.011  2.342  1.472  .994 | (.583-1.753)  (1.277-4.295)  (.786-2.755)  (.297-3.325) | .968  .006  .227  .992 |
| Change total hours informal care provision T0-T12 ^b^ | 2.006 | (1.165-3.456) | .012 | 2.577 | (1.309-5.074) | .006 | 2.726 | (1.369-5.424) | .004 |
| T0 = baseline, T12 = follow-up, n.a. = not applicable, OR = Odds Ratio, 95% CI = 95% Confidence Interval; a significance level of p < .01 is used.  ^a^ all uni- and multivariable models are adjusted for research project and intervention (yes/no/unknown).  ^b^ all uni- and multivariable models are adjusted for baseline frailty and baseline frailty domains of the care recipient, and for the baseline total hours of informal care provision a week.  ^c^ statistically significant (p <.01) in model without adjustment for relational problems at T0.  ^d^ not able to calculate due to small numbers of respondents. | | | | | | | | | |

| **Table 7: Uni- and multivariable logistic regression analyses with outcome mental health problems at T12 (N=660) ^a^** | | | | | | | | | |
| --- | --- | --- | --- | --- | --- | --- | --- | --- | --- |
| **Mental health problems** | **Univariable models** | | | **Multivariable model 1 (frailty)** | | | **Multivariable model 2 (frailty domains)** | | |
| **0=no (55%), 1=some/a lot (45%)** | **OR** | **(95% CI)** | **p** | **OR** | **(95% CI)** | **P** | **OR** | **(95% CI)** | **P** |
| **Care recipient health changes T0-T12** |  |  |  |  |  |  |  |  |  |
| Increase in frailty | 4.728 | (2.404-9.297) | .000 | 3.320^c^ | (1.452-7.591) | .004 | n.a. |  |  |
| Increase in functional limitations | 2.258 | (1.415-3.603) | .001 | n.a. |  |  | 1.710 | (.925-3.163) | .087 |
| Increase in psychological well-being | .367 | (.222-.608) | .000 | n.a. |  |  | .516^c^ | (.281-.950) | .033 |
| Increase in social functioning | .757 | (.524-1.093) | .137 | n.a. |  |  | .913 | (.585-1.425) | .689 |
| Increase in health-related quality of life | .545 | (.356-.836) | .005 | n.a. |  |  | 1.158 | (.639-2.099) | .629 |
| Increase in self-rated health | .408 | (.260-.641) | .001 | n.a. |  |  | .613 | (.325-1.157) | .131 |
|  |  |  |  |  |  |  |  |  |  |
| **Care recipient characteristics** |  |  |  |  |  |  |  |  |  |
| Age | 1.018 | (.993-1.043) | .160 | 1.026 | (.988-1.066) | .179 | 1.029 | (.989-1.070) | .158 |
| Female | .799 | (.579-1.103) | .173 | 1.246 | (.756-2.053) | .388 | 1.271 | (.765-2.111) | .354 |
|  |  |  |  |  |  |  |  |  |  |
| **Caregiver characteristics** |  |  |  |  |  |  |  |  |  |
| Age | 1.009 | (.997-1.022) | .139 | 1.006 | (.979-1.035) | .647 | 1.007 | (.979-1.036) | .635 |
| Female | 1.249 | (.894-1.744) | .192 | 1.414 | (.836-2.394) | .197 | 1.394 | (.820-2.371) | .220 |
| Mental health problems T0 (ref. no) | 7.614 | (5.356-10.824) | .000 | 8.069 | (5.479-11.882) | .000 | 7.876 | (5.304-11.695) | .000 |
|  |  |  |  |  |  |  |  |  |  |
| **Care situation characteristics** |  |  |  |  |  |  |  |  |  |
| Type of care relationship (caring for)  - Spouse  - Parent (in-law)  - Other | ref.  .775  .541 | (.549-1.094)  (.305-.959) | .148  .036 | ref.  1.371  1.352 | (.399-4.714)  (.398-4.596) | .617  .629 | ref.  1.434  1.422 | (.413-4.976)  (.417-4.845) | .570  .574 |
| Living together  - T0: no, T12: no  - T0: yes, T12: yes  - T0: no, T12: yes  - T0: yes, T12: no  - unknown/missing | ref.  1.416  ^d^  ^d^  ^d^ | (1.010-1.984) | .044 | ref.  1.903  ^d^  ^d^  ^d^ | (.678-5.337) | .221 | ref.  2.027  ^d^  ^d^  ^d^ | (.718-5.722) | .182 |
| Support other caregiver/volunteer available  - T0: no, T12: no  - T0: yes, T12: yes  - T0: no, T12: yes  - T0: yes, T12: no  - unknown/missing | ref.  1.070  1.268  1.337  .756 | (.699-1.639)  (.787-2.044)  (.809-2.209)  (.282-2.027) | .754  .329  .258  .578 | ref.  1.010  1.053  1.488  .758 | (.599-1.705)  (.591-1.877)  (.816-2.710)  (.235-2.443) | .972  .861  .195  .642 | ref.  1.009  1.023  1.476  .705 | (.589-1.729)  (.569-1.839)  (.804-2.712)  (.215-2.310) | .973  939  .209  .564 |
| Change total hours informal care provision T0-T12 ^b^ | 1.236 | (.753-2.026) | .402 | 1.506 | (.799-2.836) | .205 | 1.531 | (.806-2.910) | .193 |
| T0 = baseline, T12 = follow-up, n.a. = not applicable, OR = Odds Ratio, 95% CI = 95% Confidence Interval; a significance level of p < .01 is used.  ^a^ all uni- and multivariable models are adjusted for research project and intervention (yes/no/unknown).  ^b^ all uni- and multivariable models are adjusted for baseline frailty and baseline frailty domains of the care recipient, and for the baseline total hours of informal care provision a week.  ^c^ statistically significant (p <.01) in model without adjustment for mental health problems at T0.  ^d^ not able to calculate due to small numbers of respondents. | | | | | | | | | |

| **Table 8: Uni- and multivariable logistic regression analyses with outcome physical health problems at T12 (N=660) ^a^** | | | | | | | | | |
| --- | --- | --- | --- | --- | --- | --- | --- | --- | --- |
| **Physical health problems** | **Univariable models** | | | **Multivariable model 1 (frailty)** | | | **Multivariable model 2 (frailty domains)** | | |
| **0=no (42%), 1=some/a lot (58%)** | **OR** | **(95% CI)** | **p** | **OR** | **(95% CI)** | **p** | **OR** | **(95% CI)** | **p** |
| **Care recipient health changes T0-T12** |  |  |  |  |  |  |  |  |  |
| Increase in frailty | 3.397 | (1.740-6.633) | .000 | 3.273^c^ | (1.342-7.984) | .009 | n.a. |  |  |
| Increase in functional limitations | 2.097 | (1.315-3.342) | .002 | n.a. |  |  | 1.761 | (.915-3.386) | .090 |
| Increase in psychological well-being | .447 | (.273-.733) | .001 | n.a. |  |  | .554 | (.285-1.079) | .082 |
| Increase in social functioning | .838 | (.566-1.242) | .378 | n.a. |  |  | .971 | (.582-1.617) | .908 |
| Increase in health-related quality of life | .584 | (.379-.898) | .014 | n.a. |  |  | 1.246 | (.657-2.362) | .500 |
| Increase in self-rated health | .392 | (.228-.672) | .001 | n.a. |  |  | .528 | (.260-1.073) | .078 |
|  |  |  |  |  |  |  |  |  |  |
| **Care recipient characteristics** |  |  |  |  |  |  |  |  |  |
| Age | .999 | (.975-1.025) | .966 | .965 | (.924-1.008) | .112 | .972 | (.929-1.016) | .210 |
| Female | .692 | (.497-.966) | .030 | .798 | (.467-1.362) | .408 | .821 | (.474-1.421) | .480 |
|  |  |  |  |  |  |  |  |  |  |
| **Caregiver characteristics** |  |  |  |  |  |  |  |  |  |
| Age | 1.025 | (1.012-1.039) | .000 | 1.056^c^ | (1.022-1.093) | .001 | 1.057 | (1.022-1.093) | .001 |
| Female | 1.205 | (.861-1.688) | .277 | 1.368 | (.787-2.378) | .267 | 1.365 | (.778-2.396) | .278 |
| Physical health problems T0 (ref. no) | 13.492 | (9.178-19.833) | .000 | 12.922 | (8.506-19.631) | .000 | 13.034 | (8.507-19.970) | .000 |
|  |  |  |  |  |  |  |  |  |  |
| **Care situation characteristics** |  |  |  |  |  |  |  |  |  |
| Type of care relationship (caring for)  - Spouse  - Parent (in-law)  - Other | ref.  .659  .607 | (.462-.940)  (.34-1.067) | .021  .083 | ref.  5.522  3.367 | (1.250-24.403)  (.800-14.162) | .024  .098 | ref.  5.629  3.310 | (1.257-25.196)  (.776-14.120) | .024  .106 |
| Living together  - T0: no, T12: no  - T0: yes, T12: yes  - T0: no, T12: yes  - T0: yes, T12: no  - unknown/missing | ref.  1.682  ^d^  ^d^  ^d^ | (1.186-2.385) | .004 | ref.  1.567  ^d^  ^d^  ^d^ | (.478-5.134) | .459 | ref.  1.723  ^d^  ^d^  ^d^ | (.520-5.711) | .373 |
| Support other caregiver/volunteer available  - T0: no, T12: no  - T0: yes, T12: yes  - T0: no, T12: yes  - T0: yes, T12: no  - unknown/missing | ref.  1.424  1.085  1.460  .314 | (.913-2.221)  (.666-1.769)  (.863-2.469)  (.113-.871) | .119  .743  .159  .026 | ref.  1.294  1.206  2.015  .444 | (.718-2.334)  (.643-2.261)  (1.025-3.963)  (.131-1.501) | .391  .559  .042  .191 | ref.  1.358  1.184  2.012  .343 | (.740-2.492)  (.623-2.249)  (.997-4.057)  (.095-1.235) | .323  .607  .051  .102 |
| Change total hours informal care provision T0-T12 ^b^ | 1.676 | (1.007-2.790) | .047 | 1.956 | (.986-3.880) | .055 | 2.007 | (1.001-4.022) | .050 |
| T0 = baseline, T12 = follow-up, n.a. = not applicable, OR = Odds Ratio, 95% CI = 95% Confidence Interval; a significance level of p < .01 is used.  ^a^ all uni- and multivariable models are adjusted for research project and intervention (yes/no/unknown).  ^b^ all uni- and multivariable models are adjusted for baseline frailty and baseline frailty domains of the care recipient, and for the baseline total hours of informal care provision a week.  ^c^ statistically significant (p <.01) in model without adjustment physical health problems at T0.  ^d^ not able to calculate due to small numbers of respondents. | | | | | | | | | |

| **Table 9: Uni- and multivariable logistic regression analyses with outcome problems combining daily activities at T12 (N=660) ^a^** | | | | | | | | | |
| --- | --- | --- | --- | --- | --- | --- | --- | --- | --- |
| **Problems combining daily activities** | **Univariable models** | | | **Multivariable model 1 (frailty)** | | | **Multivariable model 2 (frailty domains)** | | |
| **0=no (57%), 1=some/a lot (43%)** | **OR** | **(95% CI)** | **p** | **OR** | **(95% CI)** | **p** | **OR** | **(95% CI)** | **p** |
| **Care recipient health changes T0-T12** |  |  |  |  |  |  |  |  |  |
| Increase in frailty | 4.008 | (2.010-7.989) | .000 | 4.627^c^ | (2.029-10.552) | .000 | n.a. |  |  |
| Increase in functional limitations | 1.968 | (1.222-3.168) | .005 | n.a. |  |  | 1.579 | (.862-2.891) | .139 |
| Increase in psychological well-being | .532 | (.325-.873) | .013 | n.a. |  |  | .617 | (.335-1.136) | .121 |
| Increase in social functioning | .874 | (.582-1.312) | .515 | n.a. |  |  | 1.158 | (.710-1.890) | .555 |
| Increase in health-related quality of life | .649 | (.423-996) | .048 | n.a. |  |  | 1.322 | (.724-2.413) | .363 |
| Increase in self-rated health | .380 | (.224-.646) | .000 | n.a. |  |  | .318^c^ | (.164-.616) | .001 |
|  |  |  |  |  |  |  |  |  |  |
| **Care recipient characteristics** |  |  |  |  |  |  |  |  |  |
| Age | 1.030 | (1.004-1.056) | .023 | 1.005 | (.968-1.044) | .782 | 1.009 | (.971-1.048) | .654 |
| Female | .794 | (.572-1.102) | .167 | 1.062 | (.645-1.751) | .812 | 1.083 | (.647-1.811) | .762 |
|  |  |  |  |  |  |  |  |  |  |
| **Caregiver characteristics** |  |  |  |  |  |  |  |  |  |
| Age | .991 | (.978-1.003) | .139 | .992 | (.965-1.020) | .567 | .991 | (.964-1.019) | .526 |
| Female | 1.753 | (1.240-2.479) | .002 | 1.740^c^ | (1.033-2.931) | .037 | 1.692^c^ | (.995-2.876) | .052 |
| Problems combining daily activities T0 (ref. no) | 6.896 | (4.836-9.833) | .000 | 6.327 | (4.333-9.240) | .000 | 6.641 | (4.499-9.803) | .000 |
|  |  |  |  |  |  |  |  |  |  |
| **Care situation characteristics** |  |  |  |  |  |  |  |  |  |
| Type of care relationship (caring for)  - Spouse  - Parent (in-law)  - Other | ref.  1.321  .660 | (.993-1.870)  (.369-1.811) | .117  .162 | ref.  1.056  .837 | (.307-3.632)  (.251-2.798) | .931  .773 | ref.  1.125  .847 | (.326-3.877)  (.252-2.850) | .852  .789 |
| Living together  - T0: no, T12: no  - T0: yes, T12: yes  - T0: no, T12: yes  - T0: yes, T12: no  - unknown/missing | ref.  .931  ^d^  ^d^  ^d^ | (.663-1.306) | .677 | ref.  1.228  ^d^  ^d^  ^d^ | (.441-3.423) | .694 | ref.  1.236  ^d^  ^d^  ^d^ | (.447-3.420) | .683 |
| Support other caregiver/volunteer available  - T0: no, T12: no  - T0: yes, T12: yes  - T0: no, T12: yes  - T0: yes, T12: no  - unknown/missing | ref.  1.911  1.187  1.182  .614 | (1.241-2.943)  (.731-1.928)  (.706-1.979)  (.221-1.706) | .003  .489  .524  .350 | ref.  1.559  1.019  1.146  .737 | (.926-2.626)  (.578-1.795)  (.629-2.089)  (.227-2.394) | .095  .949  .657  .611 | ref.  1.515  1.031  .998  .609 | (.883-2.601)  (.576-1.843)  (.541-1.842)  (.182-2.036) | .132  .919  .994  .420 |
| Change total hours informal care provision T0-T12 ^b^ | 1.409 | (.834-2.382) | .200 | 1.484 | (.784-2.809) | .225 | 1.615 | (.834-3.126) | .155 |
| T0 = baseline, T12 = follow-up, n.a. = not applicable, OR = Odds Ratio, 95% CI = 95% Confidence Interval; a significance level of p < .01 is used.  ^a^ all uni- and multivariable models are adjusted for research project and intervention (yes/no/unknown).  ^b^ all uni- and multivariable models are adjusted for baseline frailty and baseline frailty domains of the care recipient, and for the baseline total hours of informal care provision a week.  ^c^ statistically significant (p <.01) in model without adjustment for problems combining daily activities at T0.  ^d^ not able to calculate due to small numbers of respondents. | | | | | | | | | |

| **Table 10: Uni- and multivariable logistic regression analyses with outcome financial problems at T12 (N=660) ^a^** | | | | | | | | | |
| --- | --- | --- | --- | --- | --- | --- | --- | --- | --- |
| **Financial problems** | **Univariable models** | | | **Multivariable model 1 (frailty)** | | | **Multivariable model 2 (frailty domains)** | | |
| **0=no (90%), 1=some/a lot (10%)** | **OR** | **(95% CI)** | **p** | **OR** | **(95% CI)** | **p** | **OR** | **(95% CI)** | **p** |
| **Care recipient health changes T0-T12** |  |  |  |  |  |  |  |  |  |
| Increase in frailty | 2.057 | (.688-6.149) | .197 | 1.325 | (.306-5.732) | .707 | n.a. |  |  |
| Increase in functional limitations | 1.378 | (.642-2.957) | .411 | n.a. |  |  | 1.140 | (.374-3.470) | .818 |
| Increase in psychological well-being | .522 | (.224-1.217) | .132 | n.a. |  |  | .515 | (.163-1.624) | .257 |
| Increase in social functioning | .746 | (.397-1.403) | .362 | n.a. |  |  | .676 | (.302-1.511) | .339 |
| Increase in health-related quality of life | .698 | (.346-1.409) | .316 | n.a. |  |  | 2.289 | (.753-6.959) | .144 |
| Increase in self-rated health | .840 | (.370-1.905) | .670 | n.a. |  |  | .662 | (.205-2.136) | .490 |
|  |  |  |  |  |  |  |  |  |  |
| **Care recipient characteristics** |  |  |  |  |  |  |  |  |  |
| Age | 1.018 | (.978-1.061) | .381 | 1.074 | (.997-1.156) | .060 | 1.082 | (.998-1.174) | .056 |
| Female | 1.143 | (.658-1.988) | .635 | 2.359 | (.898-6.200) | .082 | 2.555 | (.947-6.896) | .064 |
|  |  |  |  |  |  |  |  |  |  |
| **Caregiver characteristics** |  |  |  |  |  |  |  |  |  |
| Age | 1.004 | (.983-1.025) | .720 | .969 | (.914-1.027) | .289 | .968 | (.909-1.030) | .305 |
| Female | .761 | (.443-1.305) | .321 | 1.347 | (.508-3.573) | .550 | 1.393 | (.510-3.806) | .518 |
| Financial problems T0 (ref. no) | 28.046 | (14.519-54.172) | .000 | 48.636 | (20.775-113.859) | .000 | 61.114 | (24.273-153.875) | .000 |
|  |  |  |  |  |  |  |  |  |  |
| **Care situation characteristics** |  |  |  |  |  |  |  |  |  |
| Type of care relationship (caring for)  - Spouse  - Parent (in-law)  - Other | ref.  .872  .366 | (.498-1.527)  (.107-1.253) | .632  .110 | ref.  .949  .523 | (.101-8.945)  (.044-6.207) | .964  .608 | ref.  .943  .563 | (.088-10.056)  (.042-7.574) | .961  .665 |
| Living together  - T0: no, T12: no  - T0: yes, T12: yes  - T0: no, T12: yes  - T0: yes, T12: no  - unknown/missing | ref.  1.684  ^d^  ^d^  ^d^ | (.954-2.975) | .072 | ref.  2.582  ^d^  ^d^  ^d^ | (.516-12.917) | .248 | ref.  2.810  ^d^  ^d^  ^d^ | (.513-15.388) | .234 |
| Support other caregiver/volunteer available  - T0: no, T12: no  - T0: yes, T12: yes  - T0: no, T12: yes  - T0: yes, T12: no  - unknown/missing | ref.  .502  ^d^  ^d^  ^d^ | (.228-1.105) | .087 | ref.  .564  ^d^  ^d^  ^d^ | (.203-1.568) | .273 | ref.  .583  ^d^  ^d^  ^d^ | (.200-1.702) | .323 |
| Change total hours informal care provision T0-T12 ^b^ | 1.198 | (.490-2.784) | .727 | 1.424 | (.453-4.474) | .545 | 1.608 | (.489-5.287) | .434 |
| T0 = baseline, T12 = follow-up, n.a. = not applicable, OR = Odds Ratio, 95% CI = 95% Confidence Interval; a significance level of p < .01 is used.  ^a^ all uni- and multivariable models are adjusted for research project and intervention (yes/no/unknown).  ^b^ all uni- and multivariable models are adjusted for baseline frailty and baseline frailty domains of the care recipient, and for the baseline total hours of informal care provision a week.  ^c^ statistically significant (p <.01) in model without financial problems at T0.  ^d^ not able to calculate due to small numbers of respondents. | | | | | | | | | |
